# Development of a novel mechanism-based glycolipid adjuvant for vaccination

**DOI:** 10.12688/f1000research.13794.1

**Published:** 2018-05-30

**Authors:** Jordana Grazziela Coelho-dos-Reis, Xiangming Li, Moriya Tsuji

**Affiliations:** 1 Centro de Pesquisas René Rachou, Fundação Oswaldo Cruz, Belo Horizonte, Minas Gerais, Brazil; 2 Aaron Diamond AIDS Research Center, Affiliate of The Rockefeller University, New York, NY, USA; 3 Sanofi, Cambridge, MA, USA

**Keywords:** glycolipid, adjuvant, natural killer T cell, CD1d, malaria vaccine, dendritic cell

## Abstract

The inability to elicit strong and durable cellular responses is a major obstacle in the development of successful vaccines, in particular those against malaria. In this regard, the generation of novel adjuvants that will potently boost cell-mediated immunity induced by candidate vaccines is helpful. We and others have found a glycolipid, called α-galactosylceramide (α-GalCer), which could be presented on CD1d expressed by antigen-presenting cells (APCs) and stimulate natural killer T (NKT) cells. This triggers the activation/maturation of APCs, particularly dendritic cells (DCs). By activating NKT cells and subsequently DCs, α-GalCer has been shown to enhance adaptive immune responses, particularly of CD8 ^+^ T cells, induced by the vaccines. More recently, we identified an analogue of α-GalCer, which can display a potent adjuvant activity in conjunction with malaria vaccines in mice and non-human primates. It is anticipated that CD1d-binding, NKT cell-stimulating glycolipids will be tested as adjuvants in humans in the near future.

## Introduction

The development of effective vaccines remains the key to eradicating many prevalent global pathogens, such as malaria. In order to develop successful vaccines against these diseases, one has to elicit powerful and long-lasting protective immunity consisting of humoral and cellular responses, as both are shown to be essential for effectively eliminating pathogens. The inability to elicit potent, durable, and protective T-cell responses, particularly CD8 ^+^ T-cell responses, has been a major obstacle in developing successful vaccines.

Many efforts to identify a new adjuvant have been undertaken in order to overcome the limitations of current vaccines ^1– 5^. An adjuvant is a compound that helps enhance immune responses elicited by vaccines. For example, a weak immunogen requires an adjuvant for the enhancement of its immunogenicity. In some cases of viral vectored vaccines, the pre-existing immunity to the vector itself has been shown to reduce their immunogenicity ^6, 7^, and hence the use of adjuvants that circumvent the pre-existing immunity might be important to augment the efficacy of the vaccines.

Antigen-presenting cells (APCs) express a non-polymorphic major histocompatibility complex (MHC) class I–like molecule, called CD1d, which presents lipids to unconventional T cells, called invariant natural killer T ( *i*NKT) cells, and stimulate them through their invariant α/β T-cell receptor (TCR) ^8– 10^. Upon stimulation, the *i*NKT cells rapidly secrete cytokines, such as interferon-gamma (IFN-γ), and, together with CD40-CD40L interactions, *i*NKT cells induce maturation and activation of APCs ^9^.

**Figure 1.**
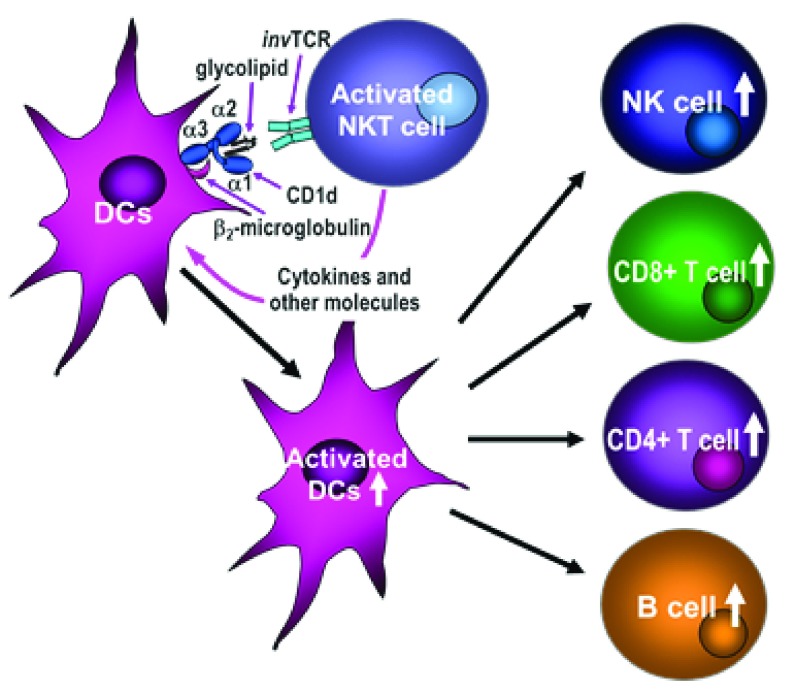
Mode of invariant natural killer T (NKT) cell activation by glycolipids and subsequent activation of various immune competent cells. Glycolipids presented by CD1d molecules activate invariant NKT cells, which in turn induce activation/maturation of dendritic cells (DCs) and subsequent activation of CD8 ^+^ T cells, and also natural killer (NK), B, and CD4 ^+^ T cells. TCR, T-cell receptor.

Alpha-galactosylceramide (α-GalCer), which is a well-known glycolipid that binds CD1d ^11– 14^, activates *i*NKT cells to rapidly produce large quantities of Th1 and Th2 cytokines and subsequently induces the activation of a cascade of various immuno-competent cells, including dendritic cells (DCs), NK cells, B cells, and CD4 ^+^ and CD8 ^+^ T cells ( Figure 1). α-GalCer has been used not only as a potential direct therapy for cancer and autoimmune and infectious diseases ^15– 24^ but also as an adjuvant to enhance the efficacy of various existing or new vaccines that include live vector vaccines ^25– 30^.

It is noteworthy that we have more recently identified a lead clinical candidate synthetic analog of α-GalCer, named 7DW8-5, which elicits the highest level of *i*NKT-cell response among 100 α-GalCer analogs tested ^31^. When we co-administered 7DW8-5 or α-GalCer intra-muscularly with a sub-optimal dose of a recombinant adenovirus expressing a major malaria antigen, the circumsporozoite protein of *Plasmodium yoelii* (PyCSP), we found that 7DW8-5 has a 100-fold higher dose-sparing effect than α-GalCer in displaying the adjuvant effect on the level of PyCSP-specific CD8 ^+^ T-cell response induced in mice upon its single immunizing dose ^31^. In this article, we will review the adjuvant effects of glycolipids, which bind CD1d and stimulate *i*NKT cells, in the context of certain vaccines.

### Identification of a potent CD1d-binding NKT-cell ligand

**Figure 2.**
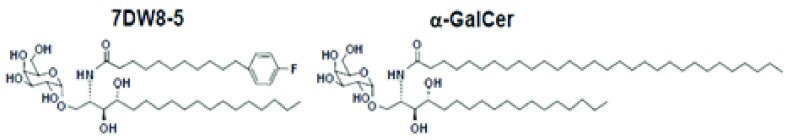
Structure of 7DW8-5 and α-galactosylceramide (α-GalCer).

From a focused glycolipid library consisting of about 100 analogs of α-GalCer, we have identified a lead candidate glycolipid, 7DW8-5, which exhibits a more potent biological activity than its parental compound, α-GalCer ^31^. The formal chemical name of 7DW8-5 is [(2S, 3S, 4R)-1-O-(α-D-galactopyranosyl)-N-(11-(4-fluorophenyl) undecanoyl)-2-amino-1,3,4-octadecanetriol)]. This analog differs from α-GalCer in that it possesses a fluorinated benzene ring at the end of a C8 length fatty acyl chain ( Figure 2). 7DW8-5 was shown to have a much higher binding affinity than α-GalCer for murine and human CD1d molecules and consequently display a stronger stimulatory activity toward *i*NKT cells and CD1d-expressing DCs. We also determined various functional properties of the two glycolipids. Table 1 contains a summary of published results comparing the properties of two glycolipids: 7DW8-5 and α-GalCer ^31– 37^.

### A potent CD1d-binding NKT-cell ligand as an adjuvant for malaria vaccines

As for its safety, when 7DW8-5 was administered together with a human malaria vaccine to non-human primates, 7DW8-5 not only exhibited a significantly potent adjuvant effect to enhance the malaria vaccine-induced T-cell responses but also displayed no systemic reactogenicity ^32^. It is noteworthy that 7DW8-5 combined with a TLR4 agonist, monophosphoryl lipid A (MPLA), displays a potent adjuvant effect and enhances the levels of antigen-specific CD8 ^+^ T-cell responses with effector memory function and protective immunity to malaria and cancer ^35^.

Most recently, we have found that after co-administration of the glycolipid with a malaria vaccine based on radiation-attenuated sporozoites (RASs) of a rodent malaria, in contrast to α-GalCer, 7DW8-5 co-localizes with murine RASs in the draining lymph nodes and induces activation/maturation of DCs that present malaria antigens. This cascade of events resulted in enhancing the levels of malaria-specific CD8 ^+^ T-cell response and ultimately the level of protective anti-malaria immunity induced by the RAS vaccine ( Table 1) ^34^. In terms of translational value, it is exciting to report that when 7DW8-5 and an adenovirus-based human malaria vaccine were co-administered to our cutting-edge humanized mice that possess functional human CD8 ^+^ T cells and *i*NKT cells ^37^, 7DW8-5 was shown to enhance not only the malaria antigen-specific human CD8 ^+^ T-cell response but also the efficacy of the vaccine ^37^. As expected, the adjuvant effect of 7DW8-5 was more potent than that of α-GalCer in humanized mice ^37^.

**Table 1.** Summary comparing the properties between 7DW8-5 and α-galactosylceramide (α-GalCer) ^31– 37^.

1. 7DW8-5 and α-GalCer possess very similar chemical structures; the only difference is the fatty acyl chain, in which a terminal fluorinated benzene ring is attached for 7DW8-5 ( Figure 2). 2. 7DW8-5 binds mouse and human CD1d molecules with a 30- to 80-fold higher affinity than α-GalCer and stimulates invariant natural killer T ( *i*NKT) cells *in vitro* with a 100-fold higher dose-sparing effect than α-GalCer. 3. 7DW8-5 induces the upregulation of major histocompatibility complex (MHC) II and CD86 expression by dendritic cells (DCs) almost two-fold higher than that induced by α-GalCer. 4. 7DW8-5, but not α-GalCer, can co-localize in the draining lymph nodes (dLNs) with mouse malaria vaccine upon co- administration by the intra-muscular route and induce activation/maturation of lymph node–resident DCs. 5. The co-localization of 7DW8-5 with a malaria vaccine in dLNs results in enhancing malaria-specific CD8 ^+^ T-cell responses and ultimately protective anti-malaria immunity induced by the vaccine. 6. 7DW8-5 can enhance malaria-specific CD8 ^+^ T-cell responses induced by a human malaria vaccine in non-human primates while displaying no systemic reactogenicity. 7. 7DW8-5 displays an adjuvant effect by enhancing protective CD8 ^+^ T-cell responses against WT1 tumor *in vivo*. 8. 7DW8-5 displays an adjuvant effect in human immune system (HIS) mice by enhancing malaria-specific human CD8 ^+^ T-cell response and protective efficacy against hybrid rodent malaria parasites that express human malaria antigen.

## Conclusions

Many candidate vaccines evaluated to date fail to achieve protection against certain human pathogens, such as malaria, and this is primarily due to their poor cellular immunogenicity. In this regard, glycolipids, which bind CD1d molecules and stimulate *i*NKT cells, have been shown to exert an adjuvant effect on liver vector-based vaccines and also to increase the level of CD8 ^+^ T-cell responses induced by the vaccines. Our proof-of-concept data on 7DW8-5 *in vitro* in parallel with normal mice, in a humanized mouse model, and in non-human primates clearly depict this potent glycolipid as a safe and effective immune-boosting adjuvant for a malaria vaccine, which could support future studies of 7DW8-5 as an adjuvant for other vaccines against HIV, cancer, and autoimmune diseases. Furthermore, it is plausible that this glycolipid adjuvant may add value when it is used in combination with existing adjuvants. Therefore, we hope to facilitate the clinical development of 7DW8-5 as a potent adjuvant to aid in the development of successful vaccines, such as a malaria vaccine, in humans in the near future.

## Abbreviations

α-GalCer, α-galactosylceramide; APC, antigen-presenting cell; DC, dendritic cell; *i*NKT, invariant natural killer T; PyCSP, circumsporozoite protein of *Plasmodium yoelii*; RAS, radiation-attenuated sporozoite
